# GC-MS and PCA Analysis of Fatty Acid Profile in Various *Ilex* Species

**DOI:** 10.3390/molecules29204833

**Published:** 2024-10-12

**Authors:** Anna Zwyrzykowska-Wodzińska, Bogdan Jarosz, Piotr Okińczyc, Jakub Szperlik, Przemysław Bąbelewski, Zdeněk Zadák, Anna Jankowska-Mąkosa, Damian Knecht

**Affiliations:** 1 Institute of Animal Breeding, Wroclaw University of Environmental and Life Sciences, Chełmońskiego 38c, 51-630 Wrocław, Poland; anna.jankowska-makosa@upwr.edu.pl (A.J.-M.); damian.knecht@upwr.edu.pl (D.K.); 2 Department of Food Chemistry and Biocatalysis, Wroclaw University of Environmental and Life Sciences, Norwida 25, 50-375 Wrocław, Poland; bogdan.jarosz@upwr.edu.pl; 3 Department of Pharmacognosy and Herbal Medicines, Wrocław Medical University, Borowska 211A, 50-556 Wrocław, Poland; piotr.okinczyc@umw.edu.pl; 4 Laboratory of Tissue Culture, Botanical Garden, Faculty of Biological Sciences, University of Wrocław, 50-234 Wrocław, Poland; jakub.szperlik@uwr.edu.pl; 5 Department of Horticulture, Wrocław University of Environmental and Life Sciences, pl. Grunwaldzki 24A, 50-363 Wrocław, Poland; 6 Department of Clinical Biochemistry and Diagnostics, Faculty of Medicine and University Hospital, Charles University, Sokolska Str. 581, 500 05 Hradec Kralove, Czech Republic; zdenek.zadak@fnhk.cz

**Keywords:** *Ilex* sp., fatty acids, PCA, correlation matrix, GC-MS

## Abstract

Natural compounds are important source of desired biological activity which helps to improve nutritional status and brings many health benefits. *Ilex paraguariensis* St. Hill. which belongs to the family *Aquifoliaceae* is a plant rich in bioactive substances (polyphenols, saponins, alkaloids) with therapeutic potential including hepatic and digestive disorders, arthritis, rheumatism, and other inflammatory diseases, obesity, hypertension, hypercholesterolemia. In terms of phytochemical research *I*. *paraguariensis* has been the subject of most intensive investigations among *Ilex* species. Therefore, we concentrated on other available *Ilex* varieties and focused on the content of fatty acids of these shrubs. The fatty acid compounds present in *Ilex* sp. samples were analyzed by GC-MS. 27 different fatty acids were identified in the extracts. The results showed that many constituents with significant commercial or medicinal importance were present in high concentrations. The primary component in all samples was α linolenic acid(18:3 Δ9,12,15). Differences of this component concentration were observed between cultivars and extensively analyzed by PCA, one- way ANOVA and Kruskal-Wallis ANOVA. Significant correlations between compound concentrations were reported.

## 1. Introduction

Approximately 400 species of plants of the holly family (*Aquifoliaceae*) are found in various parts of the world. These are mostly shrubs with evergreen leaves, but there are also species with seasonal leaves, such as holly *Ilex verticillata* [1,2].

In horticulture, there are numerous garden varieties with evergreen leaves, which were produced on purpose or developed spontaneously. They differ in growth strength, color, and shape of leaves, often notched with wavy edges and spiny teeth. Generally, the most frequently cultivated varieties are those with leaves that are evergreen, durable, leathery, dark green, and intensely shiny on the surface. The most popular taxon and cultivated species is the English holly *I. aquifolium* L., naturally occurring in Europe, from the Mediterranean to Great Britain, with over 200 known varieties, differing in growth strength, habit, shape, the color of leaves and fruits. Valuable varieties with colorful leaves include ‘Argentea Marginata’ (synonymous with ‘Argenteomarginata’)—a white-edged variety—the center of the leaf blade is dark green and the edge is white or white-cream. Other varieties with typical coloring are among the most popular, including ‘Alasca’ and ‘J.C.van Tol’. English holly comes in the form of a shrub of various sizes or a tree. In its natural environment, it grows up to 15 m tall. In European conditions, outside its natural range, it is a shrub or tree not exceeding 7 m in height, with a regularly conical crown, densely branched and abundantly foliaged [3,4,5].

Second in cultivation after the prickly holly is the Meserva holly (*I.*×*meserveae*), which was created as a result of crossing the I. rugosa naturally occurring in Japan and the English holly (*I. aquifolium*) in the early 1950s in Canada [6].

These plants have red-purple, strong, stiff shoots, sometimes with delicate, cork-like and elongated slats. Their leaves are leathery, spiny on the edge, ovoid or elliptical, 2.5–7 cm long and 1.8–3 cm wide, shiny on top. The most popular varieties in horticulture are; ‘Blue Angel’, Blue Princess’, and ‘Blue Prince’ [7].

Currently, the most valued worldwide is the South American species of holly, *I. paraguariensis*, which is widely used as a popular food and dietary supplement. It has documented pharmacological activity and is one of the valued pharmacognostic raw materials obtained from numerous plantations in South and Central America [5,7].

In folk medicine, mate has been used for the treatment of a number of diseases, including hepatic and digestive disorders, arthritis, rheumatism, and other inflammatory diseases, obesity, hypertension, and hypercholesterolemia. The potential cholesterol-lowering property suggested for mate may be due to the presence of saponins, which have the ability to form complexes with bile acids and/or cholesterol [8]. The extracts contain mainly polyphenols like chlorogenic acid, purine alkaloids (methylxanthines) such as caffeine and theobromine, flavonoids, a combination of vitamins such as vitamin A, B complex, C and E, tannins and numerous triterpenic saponins derived from ursolic acid [9,10,11,12,13]. The phenolic compounds of major importance in mate refer to caffeoyl derivatives, mainly monocaffeoyl quinic isomers and dicaffeoyl quinic isomers [11], and their content exceeds even that found for green tea, a typical ‘antioxidant’ product present on the market [12]. 

In terms of phytochemical research *I. paraguariensis* has been the subject of the most intensive investigations among *Ilex* species especially in terms of polyphenolic compounds. Therefore, we concentrated on other available *Ilex* varieties and focused on the content of fatty acids of these shrubs. Our choice of *Ilex* species was mostly dictated by their availability in Europe, including their popularity in collections of botanical gardens and nurseries. 

In the collection of decorative trees and shrubs of Wrocław University of Environmental and Life Sciences 20 taxa of shrubs of the type Ilex are grown, they are represented by *I. aquifolia* and the *I.*×*meserveae* hybrid. The basic research task is to acclimatize the type of holly and conduct research related to the use of plant material for research purposes. Meserva holly is a very promising plant because it winters perfectly, i.e., it tolerates temperature drops below −15 °C and is also promising because it has no signs of invasiveness, which in the current progressive climate changes is most valued. In the Faculty of Life Sciences and Technology, preliminary research was undertaken on the selecting of holly taxa, which have high health promoting values and perfectly acclimatize to the environmental conditions of Central Europe.

Research aimed at fatty acids is crucial, as it allows for evaluation of metabolism and elucidation of chemotypes of various taxa. It also opens the possibility to evaluate the impact of various external (e.g., weather) and internal factors (e.g., sex) on metabolism and phytochemical profile of fatty acids, which in turn allows for development of cheap sources of rare fatty acids, green chemistry approach in accordance with European Green Deal and priorities of sustainable agricultural production minimalizing environmental impact. As fatty acids play a role in plant tolerance to biotic and abiotic stresses, identification of plants characterized by beneficial phytochemical composition may allow for development of varieties suitable for recultivation of degraded or postindustrial sites [14,15]. Additionally, such varieties were tested for the first time and may be a good source of fatty acids for natural cosmetics and dietary supplements.

## 2. Results

### 2.1. Chemical Composition of Samples

The fatty acid compounds present in the *Ilex* sp. samples were analyzed by GC-MS. Up to 27 different fatty acids were identified in the extracts and shown in Table 1. Samples in the Table 1 were additionally sorted into groups obtained in principal components analysis (PCA). PCA analysis was described in next paragraph (Section 2.2. Principal components analysis—PCA). Among all identified fatty acid 15 were saturated and 12 unsaturated.

Main saturated fatty acids detected in all samples were: myristic acid (C 14:0), hexadecanoic (16:0), octadecanoic (18:0), nonadecanoic (19:0), docosanoic (22:0), and tetracosanoic (24:0). Unsaturated fatty acids found in all *Ilex* sp. are: linoleic (18:2 Δ9, 12) and α-linolenic (18:3 Δ9, 12, 15). There were also differences in the sum of saturated and unsaturated fatty acid. The highest amount of saturated fatty acids (72.79%) was observed in cultivar *Iaq*.F3 (*I. aqufolium*, female specimen 3), while the lowest amount (25.76%) in *I*×*m*.M (*I.*×*meserveae*, male specimen). Detailed results of fatty acid composition are shown in Supplementary Data, together with breakdown of saturated and unsaturated fatty acids total content. 

The primary unsaturated fatty acid in all samples was α-linolenic acid (18:3 Δ9, 12, 15). Differences of this component concentration were observed between cultivars. The highest amount was observed for cultivar *Iaq*.F5 (58.93%) and the lowest for cultivar *Iaq*.F3 (1.41%). Second abundant compound was linoleic acid (18:2 Δ9,12) with highest amount in cultivar *Iaq*.F5 and lowest in *Iaq*.F3. Opposite situation was observed for main saturated fatty acid (hexadecenoic acid, 16:0). Its highest concentration (92.48%) was exhibited for *Iaq*.F3 and the lowest (21.53%) for *I*×*m*.M. Moreover, the highest concentration of single components was also exhibited for hexadecanoic acid. 

**Figure 1:**
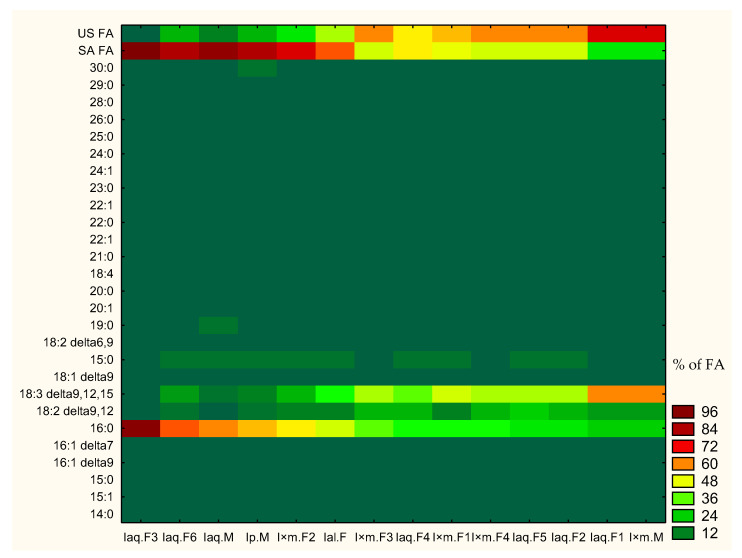
Heat map of single component presence in fatty acid profiles of *Ilex* spp. leaves. US FA—sum of unsaturated fatty acid; SA FA—sum of saturated FA; %; FA—% of fatty acid in profile.

Generally, strong difference was observed between concentrations of single components as well as sum of saturated and unsaturated fatty acid among *Ilex* sp. For this reason heat map was created (Figure 1) to show general tendencies of components concentration and changes of fatty acid profiles. Heat map was based on percent of component concentration in all fatty acid profiles. Moreover, samples were sorted in accordance with decreasing concentration of hexadecanoic acid. The heat map showed that an increase of hexadecanoic acid concentration and sum of saturated fatty acids was connected to decrease of α-linolenic acid concentration and sum of unsaturated fatty acids. Less pronounced tendency of decreasing concentration of linoleic acid was also observed, correlated to increasing concentration of hexadecanoic acid. Apart of hexadecenoic, α-linolenic and linoleic acids, the rest of singular fatty acids did not exhibited any significant tendencies. Observed tendencies were further statistically analysed in Section 2.2.2. Analyses of tendencies in fatty acid profiles in selected *Ilex* spp. are shown in Figure 1.

### 2.2. Statistical Analysis

#### 2.2.1. Principal Components Analysis (PCA)

**Figure 2:**
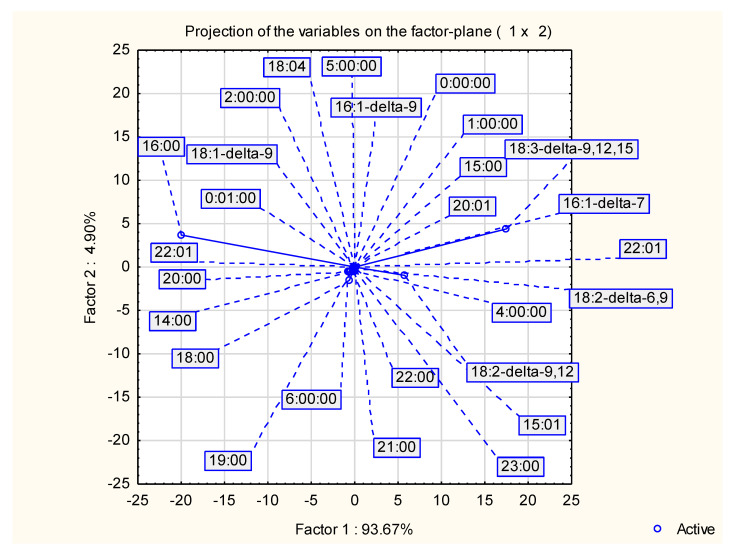
Projection of the variable on the factor plane.

**Figure 3:**
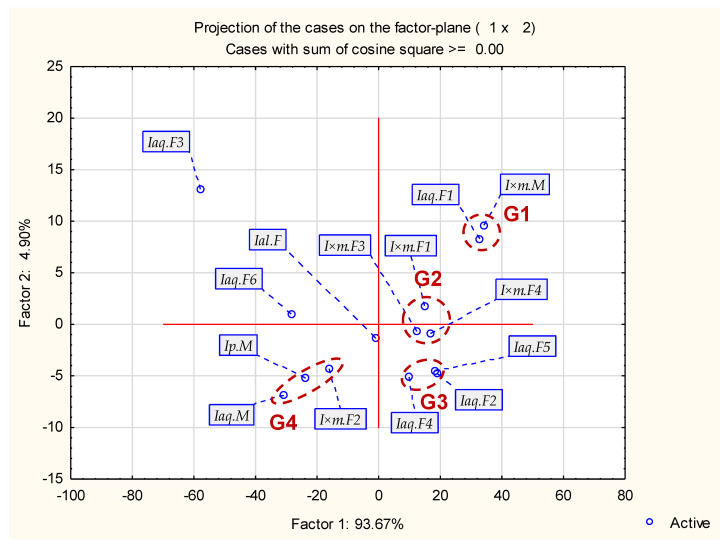
Projection of the cases on the factor plane.

Results of PCA are shown in Figure 2 (Projection of the variable on the factor plane) and Figure 3 (Projection of the cases on the factor plane). PCA exhibited, that two-factor (two principal components) model explained 98.6% of quality representation. This was sufficient to explain most of the variance in models. Factor 1 included 93.67% of representation quality, while factor 2 only 4.90%. The highest impact on factor 1 was shown by three fatty acids: 16:0 (hexadecenoic), 18:3 Δ9, 12, 15 (α-linolenic) and 18:2—Δ—9, 12 (linoleic). These three components also exhibited highest impact on Factor 2. Hexadecanoic, α-linolenic and linoleic acids were mainly responsible for projection of samples in figure Y and obtained groups.

Generally, samples may be divided in four groups shown in Figure 3. Group 1 contained *Iaq*.F1 and *I*×*m*.M. This group contained mainly α-linolenic (above 50%), lower amount of hexadecanoic acid (about 20%) and linoleic acid (about 15%). The remaining components were under 2%. Group 2 (only female specimens of *I.*×*meserveae*: *I*×*m*.F1, *I*×*m*.F3, *I*×*m*.F4) was characterized by lower amount of α-linolenic acid (about 40%) and lower amount of hexadecanoic acid (about 30%) and linoleic acid (about 11–18%). This group contains also some minor components (not present in G1). Group 3 contained only some female specimens of *I. aquifolium* (*Iaq*.F2, *Iaq*.F4 and *Iaq*.F5). This group had the most similar values of hexadecanoic acid, linoleic acid and α-linolenic acid among all groups. The last main group (G4) contained the lowest amounts of linoleic acid (3–8%) and hexadecanoic acid as main component (above 50%). α-linolenic acid shown low values (5–8%). This group contained male and female specimens (*I*×*m*.F2, *Ip*.M, *Iaq*.M). Remaining samples (female specimens–*Iaq*.F3, Ial.F and *Iaq*.F6) were classified outside or between main groups due to uncommon values of hexadecanoic (even 92.48% in *Iaq*.F3), α-linolenic and linoleic acids.

#### 2.2.2. Analyses of Tendencies in Fatty Acid Profile of Selected *Ilex* sp.

PCA has shown that fatty acids: 16:0 (hexadecanoic), 18:2 Δ9, 12 (linoleic) and 18:3 Δ9, 12, 15 (α-linolenic) had the highest impact on projecting samples on the factor plane. Moreover, heat map of these components (Figure 3) exhibited, that decreases and increases of these components were connected with each other. For these reasons, further analysis of tendencies in fatty acid profile of *Ilex* sp. was performed. It included an analysis of matrix correlation between all samples as well as ANOVA analysis.

**Figure 4:**
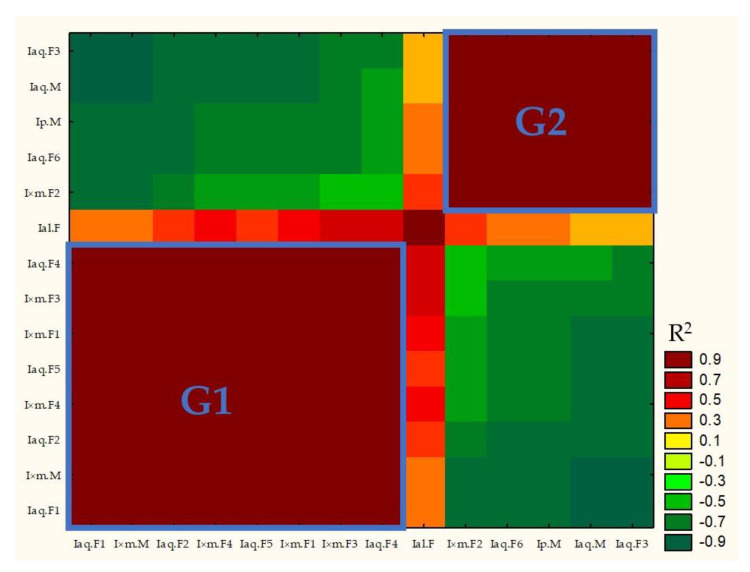
Correlation matrix R^2^ heat map.

Matrix correlation based on samples composition and its full results were presented in Supplementary Data (see Table S1. Correlation Matrix). R^2^ parameter of correlation matrix was used to build heat map (Figure 4). 

R^2^ parameter ≤ 0.506 and ≥−0.511 showed lack of correlation (*p* > 0.05, compare Figure 4 with Supplementary Table S1. Correlation matrix). Figure 4 showed that, G1 samples (left down part of graph) were positively corelated with each other and negatively correlated or not correlated with G2 samples (right top part of graph). Similar situation was observed for G2 samples. G1 samples were characterized by domination of hexadecanoic acid over α-linolenic acid, while G2 samples had higher concertation of α-linolenic than hexadecanoic acid.

Previous tests shown differences between concentration of hexadecanoic, α-linolenic and linoleic acids as well as sum of saturated and unsaturated fatty acids of samples. Therefore, one-way ANOVA and Kruskal-Walis ANOVA were used for in depth analysis. Impact of sex (male vs. female), specie (*I*. *aquifolium* vs. *I.*×*meserveae*) as well as PCA grouping were tested for the differences among mean values of hexadecanoic acid, α-linolenic, linoleic acids as well as sum of saturated and unsaturated fatty acids. Full results of all tests were provided in Supplementary Materials (Table S2 ANOVA).

In the case of PCA (Kruskal-Walis ANOVA test), statistically significant differences among mean values were shown for hexadecanoic (*p* = 0.0357) and α-linolenic acids (*p* = 0.0357) as well as sum of saturated (*p* = 0.0399) and unsaturated fatty acids (*p* = 0.0399), while linoleic acid was statistically insignificant (*p* = 0.0616). Sex (Kruskal-Walis ANOVA test) and specie (one-way ANOVA test) did not have significant impact on differences among single fatty acid and sum of saturated and unsaturated fatty acids mean values (*p* > 0.05). 

In summary, it could be suspected that leaves of selected *Ilex* spp. were primarily focused on production of hexadecanoic acid or α-linolenic acid, while production of linoleic acid was rather secondary. Production of hexadecanoic, α-linolenic and linoleic acids was also rather not directly connected with specimens sex nor specie. However, it cannot be overall excluded, that sex or specie may exhibit impact on fatty acid production. It was curious, that PCA groups did not contain female and male specimens of this same species. For this reason it may be suspected, that impact of sex as well as specie on fatty acid profile is very discreet and connected with additional factors. This hypothesis should be tested in future investigations. 

## 3. Discussion

The holly genus is represented worldwide by several species found on the continents of South America, North America, Asia and Europe. The bushes are characterized by monopodial growth with differentiation into long and short shoots. For the old world, there is only one species of holly, *Ilex aquifolium*, whose natural range extends from the Mediterranean Basin to central and southern Europe, northern Africa and Anatolia. In Europe, it grows from France, western Germany to Great Britain. This species is distinguished by its evergreen leaves and is extremely thermophilic, although it tolerates the Atlantic climate. It is characterized by high variability related to the structure of its leaves, it is dioecious and wind-pollinated, which indicates a great predisposition to crossbreeding with other hollies. So it has great predispositions for the creation of new varieties and hybrids that inherit many features of this species [1,2,3,4,5]. 

One of the hybrids that was created in Canada in the 1960s is *I.*×*meserveae*, which is a hybrid of the common holly and the tsuru holly *I. rugosa*, occurring naturally in Japan and Sakhalin. This hybrid forms low, wide-spreading shrubs, with characteristic fine, spiny-notched leaves with veins pressed into the surface of the leaf blade. It is distinguished by its similarity to the common holly because it is evergreen, dioecious and, like the common holly, produces fruit like red drupes in females. It has leathery leaves and is dioecious, producing female and male bushes. Genetic similarity of holly varieties meserva has resulted in morphological and anatomical features inherited at least 50% from the common holly and the tsuru holly, which proves that the similarity to both parent species may be easily shown by genetic tests and the content and composition of phenolic and fatty compounds. Varieties of common holly are very similar to the typical species. Meserva holly varieties, however, inherited the characteristics of common and tsuru hollies [5,6,7,16]. The research we conducted confirmed high similarity of the studied holly taxa. The reason for this may be high genetic similarity due to the fact that the studied taxa are forms of the typical species or hybrids between hollies. PCA analysis exhibited, that fatty acid profile of male and female leaves of *I.*×*meserveae* may divide them into several chemotypes. Some of them were very similar to some samples of *I. aquifolium* and *I. perneyi*, while others create their own specific groups. Similar phenomenon was observed also for *I. aquifolium*. Moreover, no male specimen of *I.*×*meserveae*, nor *I. aquifolium* did group within this same group with female specimens of this same taxa. This may suggest, that sex of some *Ilex* sp. plays discreet role in fatty acid production, but it requires further and more thorough research to prove or disprove this suspicion. 

It has been long established that fatty acids play varied and important roles in plant metabolism. Fatty acids, both free and as part of more complex lipids, perform three main roles in plants, besides some minor functions. Firstly, the same as in animals and humans, they are metabolic fuel, both in the sense of being a source of energy, as well as its storage. Secondly, they are the main component of all cell membranes and are responsible both for their correct fluidity and proper anchoring of proteins associated with lipid bilayers. Lastly, they act as gene expression regulators. Fatty acids are generally divided into saturated and unsaturated fatty acids, the proportion of which plays a crucial role in maintaining cell membrane fluidity [14]. 

The unsaturated fatty acid, namely linoleic and α-linoleic acids were the most abundant fraction of fatty acids in more than half of the analyzed *Ilex* varieties when taken together. This is interesting, as those fatty acids not only play a crucial role in maintaining cell membrane fluidity, but also in cold stress tolerance and phenolic compounds synthesis regulation and maintaining antioxidant capacity. Wang et al. have shown that introducing LuFAD2A and LuFAD3A desaturases from flax (*Linum usitatissinum* L.) to *Arabidopsis thaliana* not only increased general content of fat in seeds, but also their unsaturated acid content, namely of linoleic acid. This in turn had increased the biosynthesis of jasmonic acid which resulted in increased cold tolerance. It was also observed than when the amount of available linoleic acid and thus jasmonic acid is insufficient, plants respond to cold stress through anthocyanin accumulation [17]. As Saffaryazd et al. have reported regarding concurrent changes in the content of fatty acids, including linoleic and α-linoleic acids, and phenolic compounds during phenological growth stages of purslane (*Portulaca oleracea* L.), the role of those compounds may not always compensate for one another, as the rise and fall of content of fatty acids and phenolics was roughly following the same pattern. Changes in both groups of compounds were also shown to correlate to changes in free radical scavenging capacity, suggesting certain interplay between their functions [18].

The second most common in the majority of analyzed varieties, and the dominant one in five of them was hexadecanoic acid. It is in general the most common fatty acid in plants, animals and microorganisms. It is not surprising, as hexadecanoic acid is known to play a crucial role in stabilizing the lipid bilayer, through the formation of liquid crystals that stabilize the various enzymatic proteins associated with cell membrane. It has been noted that the content of saturated acids in general and hexadecanoic acid in particular cannot fall below certain level, at least in higher plants and algae, or those enzymes lose the environment necessary for their function. It seems that hexadecanoic acid has a role in defense against cold stress, as its level was shown to rise in response to it [19]. Increased content of hexadecanoic acid in chloroplast membranes enhances thermal stress resistance as well as the resistance to thermal inactivation of photosynthetic electron transport [20]. Additionally, the role of hexadecanoic acid in resistance to fungal infection and impact on rizosphere were recently investigated [21].

Other fatty acids in *Ilex* spp. have been universally found in much lower concentrations, only rarely exceeding 5% of total fatty acids, and even that only concerning myristic acid in vast majority of analyzed varieties. 

Myristic acid is a saturated fatty acid responsible for repair of photodamaged photosystem two in cyanobacteria, acting through its glycerolypids [22], as well plays a role in resistance to infection. Surprisingly, the role turns to be a negative one, as the presence of cinnamic, myristic and fumaric acids in tobacco root exudates was shown to induce infection by *Ralstonia solanacearum* [23].

Stearic acid seems to play a role in repair of photodamaged photosystem two together with hexadecanoic acid in cyanobacteria [24]. The role of stearic acid, together with hexadecanoic acid, oleic acid, linoleic acid and α-linoleic acid in defense against drought stress have also been researched for wheat. It seems that the changes of mutual proportions of those fatty acids in cell membrane are crucial to maintaining its fluidity and integrity during drought stress as well as post stress recovery, together with repairing of oxidative damage by ascorbate peroxidase [25]. Mutation induced stearic acid abundance was shown to change leaf and nodule anatomy and morphology in soy, albeit the results varied depending on other factors [26].

The remaining fatty acids, like behemic, lignoceric, carotic, etc. acids are generally substrates for synthesis of waxes, phospholipids, sfingolipids and other larger molecules and play little role on their own, though the role of their further metabolites in plant growth and development can be essential indeed [15,27].

Fats are an integral part of the diet of both humans and animals, providing the necessary amount of energy and playing a significant role in the proper functioning of any organism [28]. Fatty acids as an animal feed additive serve many functions, including: increasing animal productivity, improving feed utilization, affecting animal condition, but also increasing the nutritional value of feed [29]. Due to multifaceted function of fatty acid in plants, selection of *Ilex* varieties with specific profile of them may be useful in different ways. Firstly, specific profile of fatty acid in *Ilex* leaves may be used for selection of *Ilex* varieties most resistant to weather conditions. Potentially, selected varieties may be planted in difficult environmental conditions and used as source of other important secondary metabolites such as polyphenols as well as saponins. Moreover, same fatty acid may be used in food and pharmaceutical industry in different ways. For example, chosen chemotypes may be planted and used as potential sources of fatty acids. Some of them can be used in ecological farms as natural fungicides, as chemical preparations are forbidden for such. This is important in sustainable development strategies such as “from farm to fork” [30] and The European Green Deal [31]. 

Monogastric animals have a high ability to convert fatty acids supplied with food into their tissue structures [32]. Recently, the effects of feeding animals with fatty acid supplementation gain more attention in terms of beneficial improvement on digestibility, productivity and metabolism. 

Sears et al. (2020) research shows the possibility of using hexadecanoic acid in the diet of dairy cattle. These results indicate that the use of a diet enriched with hexadecanoic acid consistently improves milk yield, milk fat and protein content, and contributes to improved digestibility of neutral detergent fiber [33]. Another saturated fatty acid, determinated in *Ilex* sp., application in animal feed was presented by Odongo et al. (2007) where dairy cattle have been supplemented with myristic acid. In their study, they found positive effects of myristic acid by inhibition of the activity of methanogenesis in ruminant animals [34].

Linoleic acid (18:2 ω 6) and α-linoleic acid (C18:3, ω 6), present in *Ilex* sp., play important role in proper functioning of the body (cell membranes, skin, the immune system, the circulatory system and the nervous system). Research by Zwyrzykowska-Wodzińska et al. (2022) indicates positive effects of these acids by improving semen quality in boars [35].

Sierżant et al. (2022) indicates that diets contained high level of omega-3, improved the deposition of omega-3 fatty acid in chicken meat without adverse effects on the meat’s oxidative stability [36].

Already attempts of *Ilex* sp. administration to livestock have been made so getting to know the composition may translate into an understanding of how it works directly as a phytobiotic/supplement in the diet of animals and how it translates into a positive impact on the body.

The effect of *I. paraguariensis* supplementation on small ruminants was described in a study by Po et al. (2012). The addition of 2.5% *I. paraguariensis* increased feed intake in lambs and resulted in 25% higher rate of wool growth than animals in the control group. In another study, the inclusion of 2.5% *I. paraguariensis* in the diet of lambs changed the composition of the milk, specifically increasing fat, protein and total solids concentration, as well as affecting lower lactose content [37,38,39]. Calves with *I. paraguariensis* supplementation have lower weights and, at the same time, significantly lower plasma triglyceride concentrations, compared to the control group [40]. In addition, calves of the experimental group, showed lower plasma total protein and albumin concentrations. A significant effect was observed for total plasma antioxidant concentration (TAC), with calves drinking milk enriched with *I. paraguariensis* presenting lower levels compared to the control group.

In another experiment the effects of *I. paraguariensis* on dairy cows during lactation (experiment 1) and during the drying-out period (experiment 2) were studied. In experiment 1, the milk yield of *I. paraguariensis*-supplemented cows was significantly higher than that of the control group. In addition, the supplemented cows had a lower body weight than those in the control group, as early as the third week of the experiment. It was also observed that cows with *I. paraguariensis*-enriched diets had significantly higher concentrations of plasma advanced oxidative protein products (AOPP) than those in the control group. In experiment 2, cows supplemented with *I. paraguariensis* at 500 g/cow/day had higher milk yields and showed lower levels of the oxidative stress index than animals in the control group [41].

In their work, Hartemink et al. (2015) reported on the effect of *I. paraguariensis* supplementation on nutrient degradation. The results of this study confirmed the positive effect of *I. paraguariensis* supplementation, manifested in the degradation of fiber and protein in the rumen. The presence of *I. paraguariensis* in cows’ diets also reduced ammonia production and increased protein availability for production purposes [42].

Livestock nutrition is constantly seeking innovative and effective methods to improve health and production efficiency. *Ilex* sp. are gaining popularity as an alternative strategy to support traditional feeding methods.

Our results also indicate that PCA and ANOVA may be used in our breeding program of *Ilex* sp. as understanding of interconnectedness of various fatty acids biosynthesis could make the breeding of plants with desired fatty acid profile significantly easier. This in turn could lead to utilization of *Ilex* species not only as ornamental plants but also as a source of novelty dietary supplements, both as supplementary animal feed and potentially as dietary supplements.

Additionally, plants with beneficial lipid composition obtained through selective breeding may be more resistant to drought, cold stress, heat stress and other environmental stresses due to their changed composition of lipid bilayers, as was discussed above. Such varieties may be highly competitive in gardening, as their improved stress tolerance could significantly reduce damage and losses in adverse environmental conditions, which are becoming increasingly frequent.

## 4. Materials and Methods

### 4.1. Plant Material

The collection of trees and shrubs is located in Psary near Wrocław, where there is a Research and Training Station of Vegetables and Ornamental Plants–Psary of the Faculty of Life Sciences and Technology of the Wrocław University of Environmental and Life Sciences. The collection consists of about 1000 plants of both trees, shrubs and perennials that are used for conducting classes with students. In the collection, shrubs of the genus *Ilex* sp. are grown, planted on a clay-sandy soil mulched with shredded tree bark layer to c. 5 cm in depth. The collection serves as a place to conduct scientific research and acclimatize selected tree taxa. Plant taxa used included: female specimens (*Ilex aquifolium*–*Iaq*.F1 (variety ‘Alaska’), *Iaq*.F2 (unspecified variety, sample 1), *Iaq*.F3 (unspecified variety, sample 2), *Iaq*.F4 (variety ‘Pyramidalis Aureomarginata’), *Iaq*.F5 (variety ‘Aureomarginata’), *Iaq*.F6 (variety ‘Alaska’); *Ilex*×*meserveae*–*I*×*m*.F1 (unspecified variety, sample 1), *I*×*m*.F2 (unspecified variety, sample 1), *I*×*m*.F3 (variety ‘Blue Girl’), *I*×*m*.F4 (variety ‘Golden Girl’); *Ilex altaclarensis*–*Ial*.F (variety ‘Lawsoniana’)) and male specimens (*Ilex aquifolium*–*Ia*.M (unspecified variety); *Ilex*×*meserveae*–*I*×*m*.M; *Ilex perneyi*–*Ip*.M).

Plant material, i.e., shoots with leaves, from annual growths from a given season were collected from the bushes evenly on the range of the entire crown both from the north, south western and eastern sides evenly. Sections of annual increases were collected about 10–20 cm long. Plant material was collected on fourth of May 2017.

### 4.2. Total Lipid Extraction and Fatty Acids Analysis

The procedure used in the present study followed classic Folch method with some modifications. Briefly, 1 g of the dry leaf sample was ground in a mortar and extracted at 25 °C overnight by stirring on a rotatory shaker at 150 rpm using 25 mL of chloroform/methanol mixture (2/1, *v*/*v*). The extract was filtered through Whatman No. 4 filter paper and evaporated to dryness at 40 °C under reduced pressure. A 25 mg sample of the dry extract was refluxed with 4 mL of 1 M methanolic KOH and subsequently treated with 4 mL of BF3 diethyl etherate (Aldrich 175501, Saint Louis, MO, USA). After usual workup the resulting mixture was analyzed using a Shimadzu GC-2010P gas chromatograph coupled with GC-QP2020 mass analyzer and equipped with Phenomenex ZB-FAME column (60 m × 0.25 μm film × 0.20 mm ID). The samples were injected at 280 °C. The analyses were carried out using helium as carrier gas at flow rate of 1.0 mL/min in a split ratio of 50:1. The column was held for 2 min at 80 °C. Oven program: ramp 1: 80 °C by 3 °C/min to 180 °C, ramp 2: 8 °C/min to 260 °C (4 min). FAMEs were identified on the basis of recorded mass spectra and the retention times obtained for the standards (Supelco 37-Component FAME Mix, Shanghai, China). Presented values are the mean value of three replications [43]. Compounds were further identifird by LRI NIST comparison with literature where possible, although for some of the compounds our values are first observed for the column used.

### 4.3. Statistical Analysis

Statistical analysis was performed by Statistica 14.0.0.15 software (Tibco SofwareInc., Palo Alto, CA, USA). Tests included principal components analysis (PCA), correlation matrix analysis and one-way ANOVA.

Data input for all tests was a percentage values of total peaks area of FAME chromatogram. Substances of at least 1% of the relative area (in any sample) were used to construct the matrix. In PCA analysis, components were used as variables, while *Ilex* samples were cases. Analysis based on covariances (as SS/(N-1)) and fatty acid were used as variables.

Correlation matrix analyses included calculation of R^2^ as well as Pearson correlation parameter between all samples. *Ilex* spp. were used as variables. Heat map for correlation matrix was prepared from calculated R^2^ parameter.

One-way ANOVA analysis were used to test impact of specie on the most important fatty acid profiles (hexadecanoic, α-linolenic and linoleic acids, sum of saturated fatty acids and sum of unsaturated fatty acids). Impact of PCA projection and sex on fatty acid profiles (hexadecanoic, α-linolenic and linoleic fatty acids, sum of saturated fatty acids and sum of unsaturated fatty acids) was tested by Kruskal-Wallis ANOVA.

## 5. Conclusions

The scientific interest in *Ilex* sp. as functional food or medicinal plant may be considered recent if compared to other plant products. We have demonstrated, that *Ilex* sp. cultivars can be divided into four groups based on their fatty acids profile. Majority of the variation is caused by hexadecanoic, α-linolenic and linoleic acids, which also explains significant differences in saturated (max. 90.50%) or unsaturated (max. 73.86%) fatty acids being dominant in the profiles. Production of hexadecanoic, α-linolenic and linoleic acids was also rather not directly connected with specimens sex nor specie. Evidence seems to support the *Ilex* species as a plant with a variety of compounds that can be used for human health and animal production. Further scientific research is needed to better understand the mechanisms of action of *Ilex* sp. and confirm their positive health effects. To our knowledge our analysis is first in it’s field and opens new avenues of research for *Ilex* sp.

## Figures and Tables

**Table 1:** Fatty acid composition of investigated *Ilex* sp. leaves.

CAS	KI (Found)	KI (NIST)	R_t_	FAME	PCA G1		PCA G2			PCA G3			PCA G4			Outside Main PCA Groups		
					*Iaq*.F1	*I*×*m*.M	*I*×*m*.F1	*I*×*m*.F3	*I*×*m*.F4	*Iaq*.F2	*Iaq*.F4	*Iaq*.F5	*I*×*m*.F2	*Iaq*.M	*Ip*.M	*Iaq*.F3	*Ial*.F	*Iaq*.F6
124-10-7	2024	2037	9.27	14:00	0.74	0.74	2.48	2.31	1.94	3.22	3.27	1.59	2.4	3.34	3.99	2.8	3.14	3.06
14101-90-7	2109	2237 ^1^	11.03	15:01	0	0	0	0.19	0	1.13	0.39	0.26	0.2	0	0	0	0.34	0
7132-64-1	2125	2124	11.37	15:00	0	0	0.7	0.28	0.41	0	0.59	0.31	0.61	0	0.63	0.08	0.84	0.82
1120-25-8	2201	2237 ^1^	13.01	16:1Δ ^9^	0	0	0	0.3	0	0	0	0.35	0.29	0	0	0	0	0
56875-67-3	2205	n.r.	13.1	16:1Δ ^7^	0	0	0	0.26	0	0	0	0.17	0.18	0	0	0	0	0
112-39-0	2226	2225	13.54	16:00	21.94	21.53	31.08	32.71	28.92	25.04	31.64	26.25	50.64	59.47	55.18	92.48	41.23	62.78
112-63-0	2395	2472 ^1^	17.24	18:2 Δ ^9,12^	15.68	14.93	11.8	18.14	17.08	17.86	16.89	20.01	8.35	3.42	4.58	0.89	11	4.39
301-00-8	2402	2583 ^2^	17.39	18:3 Δ ^9,12,15^	57.11	58.93	41.06	36.82	39.9	38.39	32.31	37.9	16.55	5.61	11.24	1.41	28.44	12.67
112-62-9	2407	2433 ^1^	17.5	18:1 Δ ^9^	0	0	0.9	0	0	0	0.57	0.18	0.5	0.27	0	0	0	0
112-61-8	2427	2434 ^3^	17.93	18:00	1.86	1.38	4.76	3.28	3.59	4.15	4.64	4.7	5.96	6.89	6.57	1.46	4.77	5.35
18287-20-2	2489	n.r.	19.26	18:2 Δ ^6,9^	0	0	0	0	0	0	0	0	0.33	0	1.12	0	0	0
1731-94-8	2528	2534 ^4^	20.07	19:00	0.45	0.43	0.58	0.17	0.19	1.31	0.99	0.86	1.66	4.34	0.74	0.06	2.65	2.26
67810-35-9	2602	n.r.	21.62	20:01	0	0	0	0.41	0	0	0	0.00	0	0	0.32	0	0	0
1120-28-1	2629	n.r.	22.15	20:00	0.47	0	1.22	0.72	0.83	1.33	1.42	1.37	2.18	2.34	2.67	0.42	1.97	1.91
2566-89-4	2666	n.r.	22.9	18:04	0	0	0	0	0	0	0	0	0.3	0	0	0	0	0
6064-90-0	2729	n.r.	24.17	21:00	0	0	0.37	0.37	0.42	0.53	0.43	0.39	0.57	0.82	0.76	0	0.56	0
1120-34-9	2805	n.r.	25.65	22:01	0	0	0	0	0	0	0	0.13	0	0	0	0	0	0
929-77-1	2830	n.r.	26.13	22:00	0.75	0.72	1.37	1.3	1.4	1.49	1.94	1.37	2.06	2.73	1.93	0.26	1.59	1.28
1120-34-9	2837	2878 ^5^	26.26	22:01	0	0	0.22	0	0	0	0	0	0	0.21	0	0	0	0
2433-97-8	2931	2951 ^5^	28.02	23:00	0.25	0.42	0.73	0.67	0.64	0.96	1.03	0.78	0.58	1.02	0.84	0	0.73	0.54
2733-88-2	3007	3079 ^5^	29.42	24:01:00	0	0	0	0	0	0	0	0.1	0	0	0	0	0	0
2442-49-1	3032	3053 ^5^	29.85	24:00:00	0.38	0.36	1	0.83	0.75	1.43	1.32	0.95	1.16	1.99	1.60	0.08	1.22	0.99
55373-89-2	3133	n.r.	31.62	25:00:00	0	0	0.27	0.19	0.16	0.49	0.39	0.27	0	0.22	0.56	0	0.2	0
5802-82-4	3228	n.r.	33.24	26:00:00	0.28	0.18	0.92	0.52	0.77	1.68	0.95	0.97	2.2	2.74	0.23	0.05	0.95	0
55682-92-3	3435	n.r.	36.59	28:00:00	0.11	0.37	0.55	0.53	0.73	0.99	0.46	0.86	1.7	1.72	1.71	0	0.36	0
4082-55-7	3535	n.r.	38.15	29:00:00	0	0	0	0	0.12	0	0	0	0	0.3	0.79	0	0	0
629-83-4	3635	n.r.	39.76	30:00:00	0	0	0	0	2.14	0	0.78	0	1.57	2.58	4.54	0	0.24	3.94
			Sum of saturated FA		27.12	25.76	45.48	71.59	42.28	41.63	49.39	39.81	71.59	88.78	81.03	97.69	60.09	82.93
			Sum of unsaturated FA		72.79	73.86	53.98	26.7	56.98	57.38	50.16	59.1	26.7	9.51	17.26	2.3	39.78	17.06

Principal component group 1; **PCA G2**—Principal component group 2; **PCA G3**—Principal component group 1; **PCA G4**—Principal component group 4. **FA**—fatty acids. **Female (F) specimens abbreviations**: *Ilex aquifolium*–*Iaq*.F1 (variety ‘Alaska’), *Iaq*.F2 (unspecified variety, sample 1), *Iaq*.F3 (unspecified variety, sample 2), *Iaq*.F4 (variety ‘Pyramidalis Aureomarginata’), *Iaq*.F5 (variety ‘Aureomarginata’), *Iaq*.F6 (variety ‘Alaska’); *Ilex*×*meserveae*–*I*×*m*.F1 (unspecified variety, sample 1), *I*×*m*.F2 (unspecified variety, sample 1), *I*×*m*.F3 (variety ‘Blue Girl’), *I*×*m*.F4 (variety ‘Golden Girl’); *Ilex altaclarensis*–*Ial*.F (variety ‘Lawsoniana’). **Male (M) specimens abbreviations**: *Ilex aquifolium*–*Ia*.M (unspecified variety); *Ilex*×*meserveae*–*I*×*m*.M; *Ilex perneyi*–*Ip*.M. ^1^ column: ZB-Wax (30 m); ^2^ column: Innowax FSC (60 m); ^3^ carrier gas: N2; ^4^ column DB-Wax (15 m/0.53 mm/1 μm); ^5^ isothermal column: DB-Wax (25 m/0.25 mm/0.22 μm); n.r.—not reported (NIST), Δ—position of double bond in carbon chain of fatty acid.

## Data Availability

Research data is available from authors.

## Supplementary Materials

The following are available online at https://www.mdpi.com/article/10.3390/molecules29204833/s1, Table S1: Correlation Matrix, Table S2: ANOVA.
